# Evaluation of an Albumin-Binding Domain Protein Fused to Recombinant Human IL-2 and Its Effects on the Bioactivity and Serum Half-Life of the Cytokine

**DOI:** 10.18869/acadpub.ibj.21.2.77

**Published:** 2017-03

**Authors:** Elham Adabi, Fateme Saebi, Amin Moradi Hasan-Abad, Ladan Teimoori-Toolabi, Gholam Ali Kardar

**Affiliations:** 1 Department of Medical Biotechnology, School of Advanced Technologies in Medicine, Tehran University of Medical Sciences, Tehran, Iran; 2 Immunology, Asthma and Allergy Research Institute, Tehran University of Medical Sciences, Tehran, Iran; 3 Molecular Medicine Department, Pasteur Institute of Iran, Tehran, Iran

**Keywords:** Albumin, Albumin-binding domain, Bioactivity, Fusion protein, Interleukin 2

## Abstract

**Background:**

Cancer immunotherapy is a promising strategy for cancer treatment. In this strategy, the immune system is triggered to destroy cancer cells. IL-2 is an important factor in passive cancer immunotherapy that helps modulating some important immune functions. One of the IL-2 limitations is low serum half-life; therefore, repetitive high doses of the injections are required to maintain effective concentrations. High-dose IL-2 therapy results in severe side effects; thus, improvement of its serum half-life would provide therapeutic benefits.

**Methods:**

We have investigated a strategy that is able to utilize an albumin-binding domain (ABD) from streptococcal protein G. In this strategy, the fusion protein ABD-rIL-2 binds to serum albumin, which results in improvement of the IL-2 serum half-life. PET26b+ plasmid was used as an expression vector, which encoded rIL-2 and ABD-rIL-2, both fused to pelB secretion signal under the control of the strong bacteriophage T7 promoter. The constructs were expressed in *E. coli* *Rosetta* (DE3), and the recombinant proteins were purified from periplasmic fractions.

**Results:**

The analysis of *in vitro* bioactivity proved that the fusion of ABD to rIL-2 does not interfere with its bioactivity. ABD-rIL-2 fusion protein indicated higher serum half-life compared to rIL-2, when it was tested in the BALB/c mice.

**Conclusion:**

The current study provides an alternative strategy to extend the half-life and improve pharmacokinetic properties of rIL-2 without reducing its bioactivity *in vitro*.

## INTRODUCTION

A mature human IL-2 molecule is a 133-amino-acid (aa) cytokine that is released primarily by activated T cells and regulates a wide range of immune responses such as differentiation, maturation, and activation of T cells, B cells, natural killer cells, as well as macrophages[1]. IL-2, as a passive cancer immunotherapy agent, has attracted attention by exhibiting some antitumor activities both as a single agent and as a complement to other therapeutic approaches[2].

Passive immunotherapy is a strategy that activates the immune system to recognize and destroy the tumor cells, mainly via the activation of the tumor-reactive T cells[3]. The high dose of IL-2 injection has been used to mediate the regression of metastatic melanomas and renal cell carcinomas[2,4]. A human recombinant form of IL-2, commercially available as Aldesleukin (Proleukin), is produced in *E. coli*. Aldesleukin has some modifications in aa sequence and has been approved to treat adults with metastatic melanoma and renal cell carcinoma. It also differs from native human IL-2 in three ways. First, it is not glycosylated since it is produced in *E. coli*. Second, its N-terminal alanine has been deleted, and third, its molecule has a cysteine to serine substitution at aa position 125, in order to reduce aggregation[5]. Both *in vitro* and *in vivo* experiments have shown that Aldesleukin possesses the biological activities similar to native human IL-2[6]. Aldesleukin has a low serum half-life of 13 to 85 minutes that is mainly attributed to its small size, allowing rapid renal clearance as well as the lack of recycling processes mediated by the neonatal fragment crystallizable receptor (FcRn). Due to its high clearance rate, repetitive high dose of injections is often required to achieve acceptable therapeutic effects, which may be associated with the cause of severe toxicity.

Vascular leak syndrome, a hypovolemic state and fluid accumulation in the extra vascular space, is the most common adverse effect of rIL-2 toxicity[7]. Thus, a method of prolonging rIL-2 *in vivo* persistence would be desirable. Several attempts such as binding IL-2 to polyethylene glycol (PEG) chains as well as genetically fusing it to human serum albumin (HSA) molecule have been carried out to improve its half-life[8]. Despite the advantages of these attempts, immunogenicity and antibody formation still remain as the major drawbacks[9]. Fusion of therapeutic proteins to albumin-binding sequences is a general strategy to improve their pharmacokinetics[10]. Albumin-binding domain (ABD) 3 of streptococcal protein G is a natural high affinity ABD consisting of 46 aa. Biacore experiments determined its HSA-binding affinity to have a kDa of ~4 nM[11]. We hypothesized that a new strategy to extend IL-2 circulation half-life would improve its immunostimulatory potency. In the present study, we applied the genetic fusion of the ABD3 domain of streptococcal protein G to rIL-2 and expressed the recombinant fusion protein in *E. coli*. The ABD-rIL-2 fusion protein demonstrated similar bioactivity compared with rIL-2 and also an extended half-life.

## MATERIALS AND METHODS

### Microorganism strains and plasmid

DH5α and *Rosetta* (DE3) strains of *E. coli* were used as hosts for cloning and expression procedures, respectively. Rosetta strains were BL21 derivatives and optimized for the expression of eukaryotic proteins containing rare codons in *E. coli*. This strain supplies transfer RNAs for AGG, AGA, AUA, CUA, CCC, and GGA codons on a compatible chloramphenicol-resistant plasmid and carries a chromosomal copy of the T7 RNA polymerase gene under the control of the lacUV5 promoter. pET26b+ (Novagen, USA) was used as rIL-2 (Aldesleukin) and ABD-rIL-2 expression plasmids.

### Expression vectors

IL-2 gene sequence was modified with first alanine deletion and the substitution of serine to cysteine at position 125 and then with N-terminal of IL-2 linked by five-aa linker (GGGGS) to the C-terminal of ABD. This transgene was cloned in expression vector pET26b+ including the pelB leader sequence (pET26b+-ABD-rIL-2). This construct was purchased from GenScript (USA). Plasmid-encoding rIL-2 was constructed by the following method. pET26b+-ABD-rIL-2 vector was used as a template, and rIL-2 gene was obtained by PCR amplification using specific forward (CCCCTACTTCAAGTTCTACAAAG) and reverse primers (TTTAAGCTTTTAAG TCAGTGTT GAG), which added a 5’ *Msc*I and 3’ *Hind*III restriction sites at the ends of rIL-2. The PCR reactions were performed with Taq DNA polymerase as follows: 94°C for 5 min, 94°C for 30 s, 50°C for 30 s, 72°C for 1 min, repeat for 30 cycles; 72°C for 8 min, 4°C hold. The PCR product containing rIL-2 fragment was purified by a high pure PCR product purification kit (Roche, Germany), and restriction fragments were cloned into the *Msc*I and *Hind*III sites of the bacterial expression vector pET26b+. The resulting vectors, pET26b+-pelB-rIL-2 and pET26b+-pelB-ABD-rIL-2, contained an N-terminal pelB leader sequence in order to enable periplasmic secretion via the sec-translocation machinery. Plasmid verification was performed by restriction analysis and then further sequenced. Each plasmid was amplified in *E. coli* (DH5α) and purified by using a plasmid purification kit (Roche, Germany). In this work, we cloned the gene sequence of Aldesleukin with mentioned modifications into pET26b+, and rIL-2 used throughout the text was referred to as Aldesleukin.

### Protein production and extraction

*E. coli* strain Rosetta (DE3) was used to express rIL-2 and ABD-rIL-2 in their periplasm. Cells were successfully transformed using CaCl_2_ and heat shock treatment. A single colony was selected and cultured in 10 ml of Luria broth (LB) containing 50 µg/ml kanamycin and 34 µg/ml chloramphenicol at 37ºC overnight. Then 1 ml of culture media was used to inoculate into 300 ml LB medium. The same concentration of the mentioned antibiotics was added to the main culture and cultivation carried out at 220 rpm at 37ºC until an optical density between 0.9–1 at OD 600 nm was reached. Thereafter, the culture was cooled on ice, isopropyl-b-D-thiogalacto-pyranoside was added to the final concentration of 1 mM, and the protein expression was carried out at 25°C for 16 hours. Bacterial cultures were then centrifuged at 4000 ×g for 10 min, and protein extraction was carried out by freeze-thawing method[12]. Briefly, the pelleted cells were frozen at -70°C for 8 min. The samples were thawed by transferring to an ice water bath for 15 min. The freeze-thawing procedure was repeated for four times, and the cells were then suspended in Tris-HCl buffer (30 mM) containing anti-protease cocktail (Roche, Germany). The mixture was placed in the ice water bath for additional 30 min. The samples were centrifuged at 20,000 ×g at 4°C for 30 min. The supernatant containing the expressed protein was carefully removed from the pellet. In addition, a two-step ultrafiltration with two different molecular weight cut-off ultrafiltration devices (10 and 30 kDa) (Sartorius, USA) were used. In this method, we fractionated our protein mixture and semi-purified it. Moreover, the majority of potential endotoxins were removed, and the remaining salts and contaminants were cleared through membrane. The *in vivo* results indicated that the protein solution was nearly free of endotoxins. After purification, the rIL-2 concentration was measured by ELISA and confirmed by Western blotting.

### SDS-PAGE and Western blot analysis

The extracted protein solutions were evaluated by 10% SDS-PAGE as described by Laemmli[13]. For Western blotting, proteins were electrophoretically transferred to polyvinylidene difluoride membranes (PVDF, Roche, Germany). PVDF membranes, which were loaded with the transferred proteins, were blocked with 5% skimmed milk dissolved in PBS containing 0.05% Tween-20. As the primary antibody, a 1:1000 dilution of mouse anti-human IL-2 was used. Biotinylated IL-2 was diluted 1:1000 served as the secondary antibody, and streptavidin-horseradish peroxidase was also added to the antibody complex. The complex was developed by the addition of the horseradish peroxidase substrate diaminobenzidine (DAB, Roche, Germany).

### Enzyme-linked immunosorbent assay

An ELISA technique was used to quantify the amount of expressed recombinant-protein present in soluble extracts based on the protocol of a standard IL-2 ELISA kit (R&D systems, USA). The concentrations of expressed proteins in the extracted samples were determined from a standard curve.

### Bioactivity assay of ABD-rIL-2 and rIL-2 *in vitro*

In order to analyze the ability of the fusion protein to stimulate cell proliferation and compare its biological activity with rIL-2, proliferation assays were performed on peripheral blood mononuclear cells (PBMCs)[14]. PBMCs were separated from human whole blood by ficoll and gradient centrifugation. The cells were cultured overnight in complete RPMI-1640 medium supplemented with 10% heat-inactivated fetal bovine serum and 1× penicillin-streptomycin antibiotic solution. On the next day, peripheral blood lymphocytes were collected from non-adherent cells and cultured in a 96-well plate. Each well was seeded with 2×10^5^ cells in complete medium in a total volume of 200 µl per well. After 24 hours, the cells were treated by two different concentrations of 1 and 1.5 ng/ml for both rIL-2 and ABD-rIL-2 in triplicates, as well as two concentrations of 1 and 2 µg/ml of phytohemagglutinin as the control. After 72 hours, 20 µl of 50 mg/ml methylthiazolyldiphenyl-tetrazolium bromide stock solution (Sigma, US) was added, and the cells were incubated for three hours. Afterwards, the solvent, methylthiazolyl-diphenyl-tetrazolium bromide, was added, and the wells were measured at 595 nm.

### Pharmacokinetic studies *in vivo*

The pharmacokinetic behavior of ABD-rIL-2 and rIL-2 was evaluated in 10-week old female BALB/c mice (about 19-21 g), which were purchased from Pharmacology Animal Lab. (TUMS, Tehran, Iran). The animals were housed and treated according to protocols approved by the Animal Care Committee of the Tehran University of Medical Sciences. Groups of 3 female BALB/c mice were injected i.v. with ABD-rIL-2 or rIL-2, at a single dose of 3 µg/kg weight. For measurement of serum drug concentrations, blood samples were periodically collected from each mouse, up to nine time points, ranging from 0 to 54 h. Serum samples were analyzed by using a commercially available IL-2 ELISA kit. Concentrations of rIL-2 or ABD-rIL-2 in the serum samples were determined using a standard curve. Results were expressed as an average of triplicate samples plus or minus standard error. The first sampling at 5 minutes was considered as zero time with concentration of 100%.

### Statistical analysis

All statistical analyses were performed using SPSS 20 software. Differences between means, which were analyzed by student’s *t*-test, and the values of *P*<0.05 were considered statistically significant. GraphPad Prism 6.07 software was used to draw graph. Pharmacokinetic factors were calculated using Excel. The mean values ± standard deviations (SD) were used to express the results.

## RESULTS

### Vectors construction

The gene *ABD-(G4S)-rIL-2* was successfully cloned into *Msc*I and *Hind*III restriction sites of pET-26b+. The *rIL-2* gene was successfully amplified using specific primers (~ 400 bp) and cloned into pET26b+ at the *Msc*I and *Hind*III restriction sites. The full lengths of *ABD-rIL-2* and *rIL-2* were confirmed by sequencing (data not shown).

### Protein expression and characterization

After 16 hours of induction with 1 mM isopropyl-β-D-thiogalactopyranoside at 25°C, both rIL-2 and ABD-rIL-2 were extracted by freezing-thawing procedure. ELISA was used to determine the protein concentration. The expression yields were 38.154 µg/L of culture for *rIL-2* and 33.422 µg/L for *ABD-rIL-2*.

### SDS-PAGE and Western blotting

**Fig. 1:**
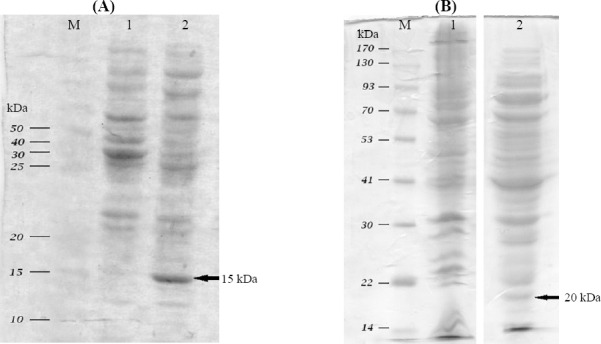
SDS-PAGE analysis of (A) extracted proteins (B) and ABD-rIL-2. M, protein ladder; Lane 1, negative control; Lane 2, extracted proteins. Arrows show the overexpressed protein band (A) and expressed protein ABD-rIL-2 (B).

**Fig.2:**
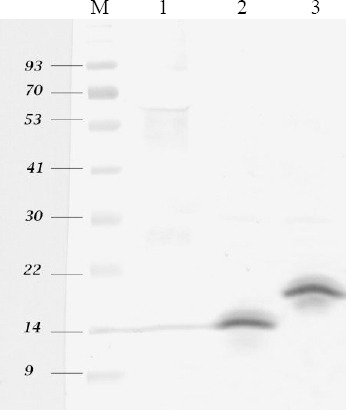
Western blot analysis of intended expressed proteins. M, protein ladder; Lane 1, negative control; Lane 2, rIL-2; Lane 3, ABD-rIL-2

The expressed ABD-(G4S)-rIL-2 fusion protein was composed of 183 aa residues with a calculated mass of 20.692 kDa. ABD-rIL-2 was ~5.7 kDa larger than rIL-2. SDS-PAGE and immunoblot analysis showed a single protein band for rIL-2 and ABD-rIL-2 at approximately 15 and 20 kDa, respectively (Fig. 1). Under reducing SDS-PAGE, the observed molecular weights were in agreement with the expected molecular weights of rIL-2 and ABD-rIL-2, indicating a proper cleavage of the pelB signal peptides on secretion into the *E. coli* periplasm. Furthermore, Western blot analysis confirmed the specificity of the observed bands in SDS-PAGE (Fig. 2).

### Bioactivity assay

**Fig. 3:**
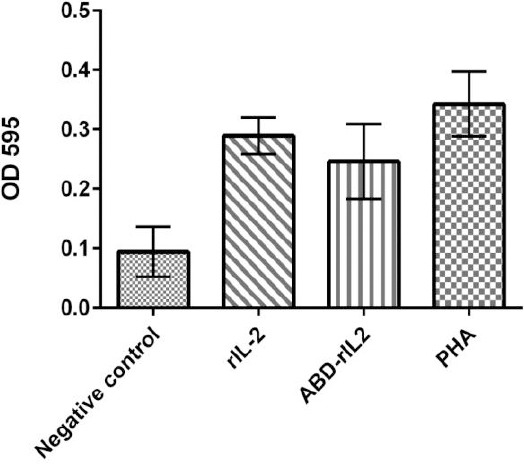
Effect of rIL-2 and ABD-IL-2 on proliferation of peripheral blood mononuclear cells. PHA (phyhaemagglutinin). The results were expressed as the mean±SEM (*P*<0.05).

Our data indicated no significant reduction in the activity of rIL-2 after fusion to ABD. Both rIL-2 and ABD-rIL-2 demonstrated approximately similar bioactivity on PBMCs proliferation (*P*=0.162) (Fig. 3). ABD-rIL-2 possessed an activity that was 85% higher than the rIL-2.

### The half-lives of ABD-rIL-2 and rIL-2

**Fig. 4:**
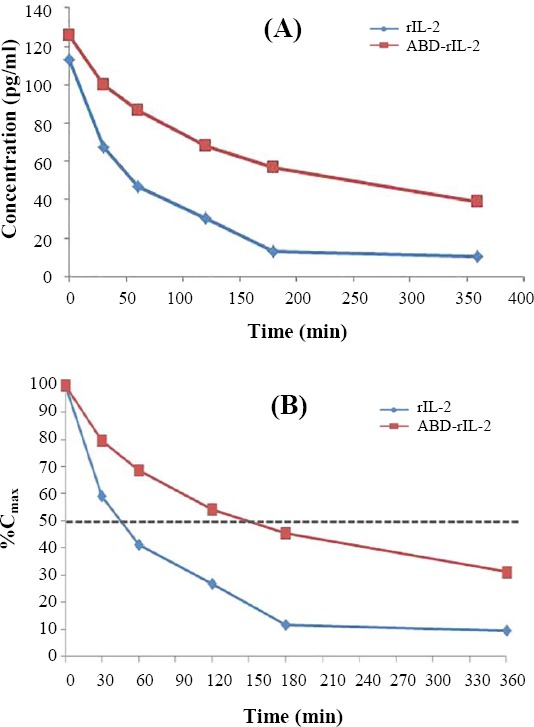
The comparison of the pharmacokinetic profile of rIL-2 and ABD-rIL-2 following i.v. administration of 3000 ng/kg in BALB/c mice. (A) Time courses of the concentrations of ABD-rIL-2 and rIL-2 in the serum. The concentrations were measured by ELISA using anti-mouse hIL-2 antibody; (B) Percentage concentration observed in mice serum vs. time.

The ABD-rIL-2 and rIL-2 data after i.v. injection are shown in Figure 4, and calculated pharmacokinetic parameters are summarized in Table 1. Following i.v. injection, the terminal half-life of rIL-2 was 46±0.2 min, which was in the range of the half-life reported for Aldesleukin (13-85 min). However, ABD-rIL-2 was presented a prolonged circulation time with an increase in its terminal half-life to 150±1.3 min, with a maximum serum concentration of 11.335±0.22 ng/ml. The clearance of ABD-rIL-2 was 77.432.3 ml/h/kg, while rIL-2 was cleared about 2.27 fold more quickly at 176.41±4 ml/h/kg after i.v. injection.

**Table 1:** Pharmacokinetic parameters of rIL-2 and ABD-rIL-2 after intravenous injection into mice

Parameter	rIL-2	ABD-rIL-2
Dose (ng/kg)	3000	3000
AUC (ng×h/mL)	17.005±0.4	38.740±1.70
CL (ml/h/kg)	176.410±4.0	77.430±2.30
t_1/2, term_ (min)	46.000±0.2	150.000±1.30
C_max_ (ng/mL)	12.580±0.8	11.335±0.22

AUC, area under (the serum concentration) curve. Which was calculated by integration to 0-360 min; C, clearance; t1/2, term, terminal half-life; C_max,_ maximal serum concentration. The data are shown as the mean±SEM.

## DISCUSSION

In the current study, *in vitro* analysis result indicated that ABD-rIL-2 fusion protein is capable of proliferating PBMCs with no meaningful difference in its bioactivity compared to rIL-2, while in Melder *et al*.[15] study, the fusion of HSA to rIL-2 showed a significant reduction in its bioactivity. This observation could be attributed to fusing rIL-2 to the high molecular weight HSA protein, resulting in rIL-2-HSA fusion protein to be five fold larger than rIL-2, while ABD-rIL-2 fusion protein is only 5 kDa larger than that. Our data confirms that this small protein domain does not interfere with rIL-2 bioactivity. The comparison of ABD-rIL-2 and rIL-2 pharmacokinetic properties revealed 2.27 fold difference in drug exposure of the body or area under the curve, and ABD-rIL-2 showed prolonged circulation half-life in mice by a factor of 3.26 compared to rIL-2. This finding indicates that ABD-rIL-2 binds to mouse serum albumin non-covalently and benefits from favorable pharmacokinetic properties of albumin to achieve prolonged circulation time. This prolonged circulation time is most likely caused by reduced renal clearance through an increase in the size of molecule and FcRn-mediated recycling effects of ABD-rIL-2 albumin complexes. The interaction of albumin with FcRn results in the activation of intracellular rescue pathway that creates protein reservoir, which also protects protein from a lysosomal degradation and subsequently recycling proteins to the extracellular space[16].

Studies have revealed that HSA-binding region of streptococcal protein G is a 46-residue triple α-helical structure that the binding site of ABD toward HSA is mainly located on the second helix; these findings were confirmed with an alanine-scanning procedure[17,18]. A five aa linker was used to genetically fuse C-terminal of ABD to N-terminal of IL-2 in order to keep the functional helix of two molecules far enough to avoid any interference in their receptor binding. HSA consists of three domains, and the FcRn-binding site resides in domain III and a part of the domain II of albumin. ABD binds to the domain II of HSA, thus ABD-binding should not be expected to interfere with FcRn-mediated recycling since they occur at different sites on an albumin molecule[19,20]. This issue has also been proven by ELISA and surface plasmon resonance for an ABD fusion to an anti-HER2 affibody molecule[21]. The contribution of FcRn in prolonging the serum half-life of ABD fusion proteins was confirmed by using knockout mice and also confirmed that the bound ABD does not interfere with the FcRn binding of albumin[22]. Furthermore, affinity measurements indicate that fusion of ABD to bispecific single-chain diabody (scDb CEACD3) does not reduce affinity for albumin. Rather the half-life of an scDb-ABD fusion protein became similar to the scDb-HSA fusion protein and a PEGylated scDb derivative[23]; Moreover, the prolonged half-life led to an increased accumulation in the target tumors compared with a PEGylated scDb, which indicates a facilitated tissue penetration of the scDb-ABD fusion protein[22]. ABD fusion bispecific affibody targeting HER2 and HER3 has been demonstrated simultaneous binding to three target proteins (HER2, HER3, and albumin) and improved circulatory half-life[24]. In one study, the anti-HER3 Affibody® molecule has been fused with an ABD in order to increase the serum half-life[5].

Being concerned about the risk of immunogenicity of ABD due to its bacterial origin, for therapeutic applications especially involving repeated injections, was successfully addressed by an engineered ABD. ABD has been unimmunized by removing T-cell epitopes located on the molecule to resemble the one being currently clinically evaluated ABD094[10]. Beside, the long serum half-life of albumin that attracts attention as an approach to improve the pharmacokinetic profile of therapeutic proteins, and albumin has been indicated to have a high accumulation in tumors as a result of enhanced vascular permeability of tumors and increase the retention of albumin in tumor interstitium[25,26]. This findings has been validated by radiolabeled or dye-conjugated albumins that have been shown to have high uptake into tumors[27]. Based on this property, HAS is considered as a delivery system for drug transference to tumor tissue[28,29]. Hence, it is proposed that ABD-rIL-2 will bind to albumin and accumulates inside tumor and induces recruitment of cytotoxic T cells to the tumor sites.

In conclusion, the high affinity of ABD fusion proteins to HSA results in improving the pharmacokinetic without reducing the bioactivity of the fusion partner. The possibility of target therapy through binding to HAS makes ABD approach as an ideal strategy to improve the pharmacokinetics of therapeutic proteins in like IL-2 in humans.
